# Supplementary material for: Preclinical Application of Reduced Manipulated Processing Strategy to Collect Transplantable Hepatocytes: A Pilot and Feasibility Study
Source: J Pers Med. 2021 Apr 21;11(5):326. doi: 10.3390/jpm11050326 (PMC8143084; doi:10.3390/jpm11050326)

Figure S1. Collagenase digestion of partial porcine liver. (A) Under collagenase perfusion through catheter into a portal vein branch. (B) 30 min after perfusion. The cell viability of hepatocytes was 91%.

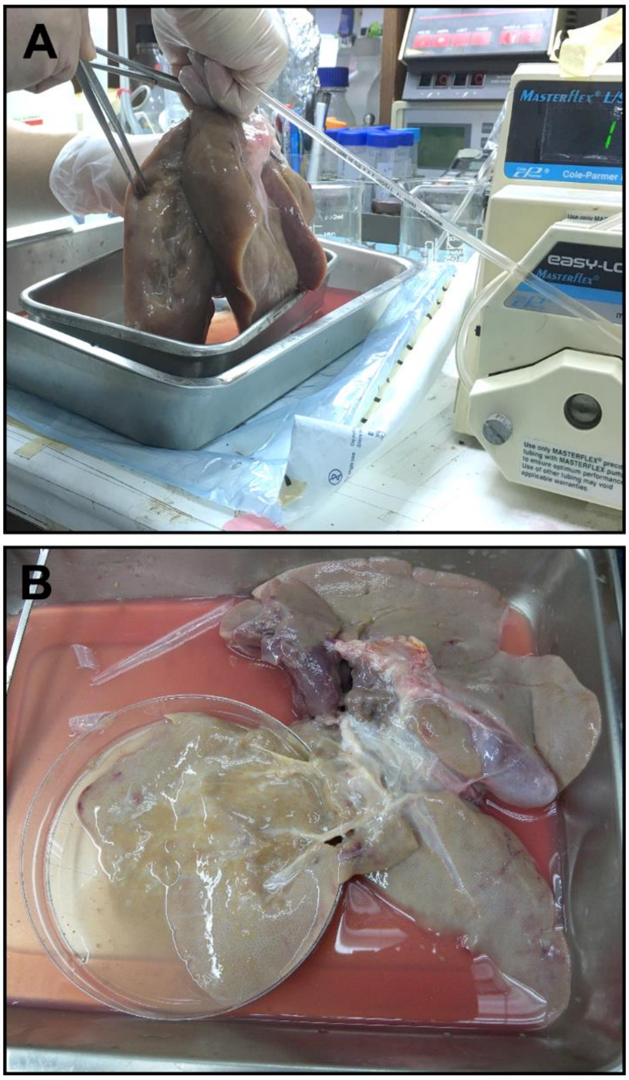

Figure S2. Cholangiocytes (marked by CK-19) and sinusoidal endothelial cells (marked by SE-1) in cellular products immediately after isolation of rat livers. Scale bar: 100 μm.

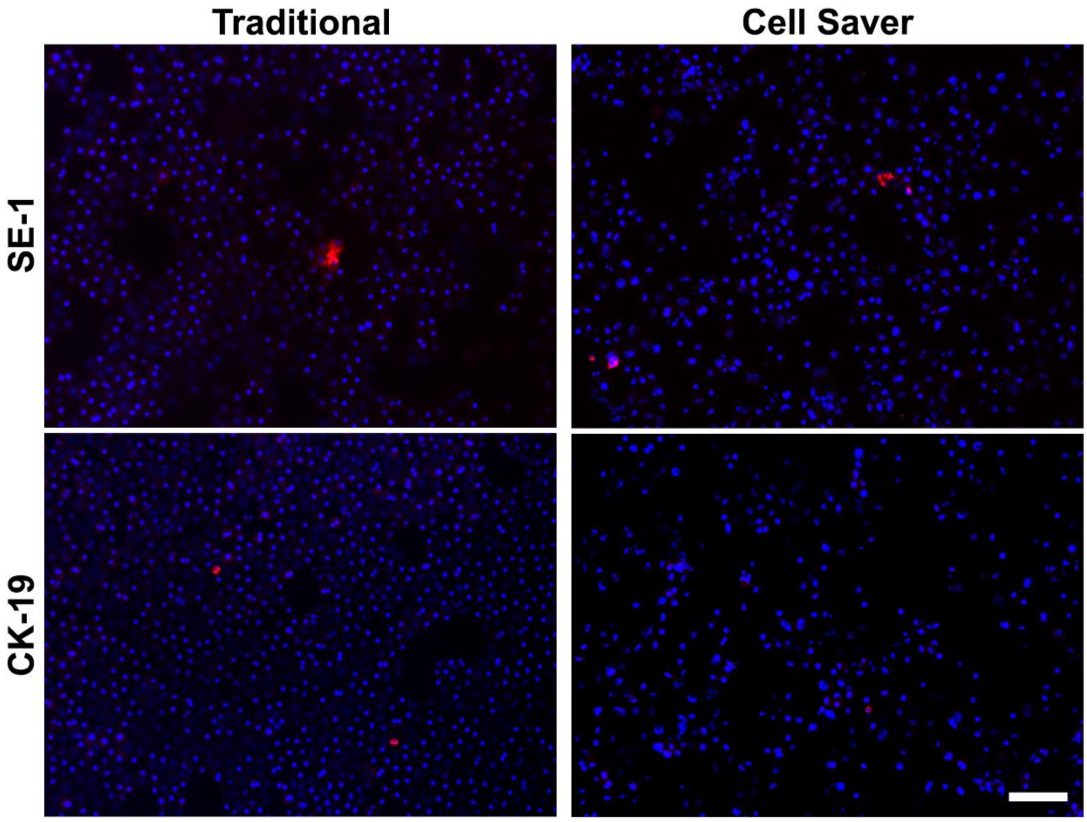

Figure S3. Isolated porcine hepatocytes obtained using the traditional method were confirmed based on albumin and cytochrome P450 3A4 (CYP3A4) expression. Scale bar: 100 μm.

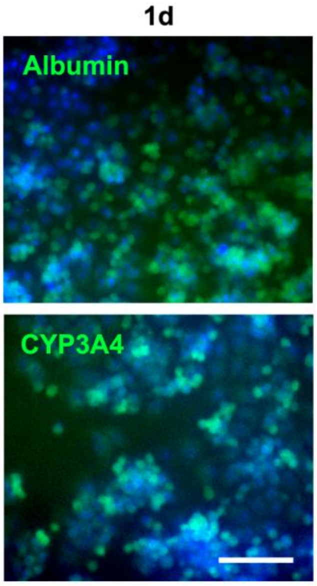

Figure S4. Experimental design for hepatocyte transplantation.

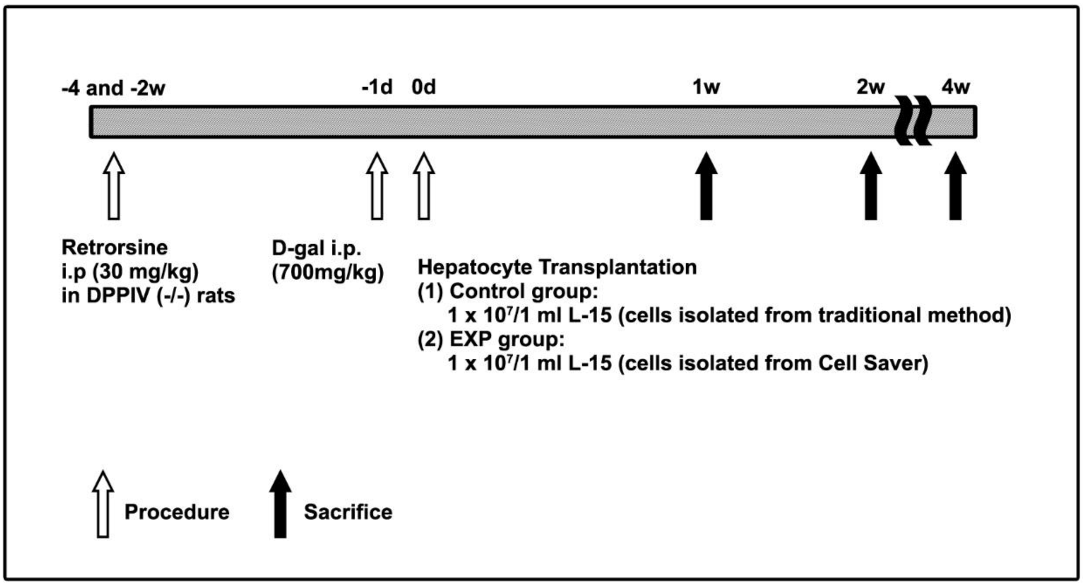

Supplement: Supplementary file 1 [file jpm-11-00326-s001.zip › jpm-1166048-supplementary.pdf]
